# Should Intelligence Tests Be Speeded or Unspeeded? A Brief Review of the Effects of Time Pressure on Response Processes and an Experimental Study with Raven’s Matrices

**DOI:** 10.3390/jintelligence11060120

**Published:** 2023-06-13

**Authors:** Corentin Gonthier

**Affiliations:** Nantes Université, Laboratoire de Psychologie des Pays de la Loire (LPPL UR 4638), Chemin de la Censive du Tertre, 44312 Nantes, France; corentin.gonthier@univ-nantes.fr

**Keywords:** intelligence, time pressure, Raven’s Advanced Progressive Matrices (APM), mental speed, item response times

## Abstract

Intelligence tests are often performed under time constraints for practical reasons, but the effects of time pressure on reasoning performance are poorly understood. The first part of this work provides a brief review of major expected effects of time pressure, which includes forcing participants to skip items, convoking a mental speed factor, constraining response times, qualitatively altering cognitive processing, affecting anxiety and motivation, and interacting with individual differences. The second part presents data collected with Raven’s matrices under three conditions of speededness to provide further insight into the complex effects of time pressure, with three major findings. First, even mild time pressure (with enough time available for all participants to complete the task at a leisurely pace) induced speeding throughout the whole task, starting with the very first item, and participants sped up more than was actually required. Second, time pressure came with lower confidence and poorer strategy use and a substantial decrease of accuracy (*d* = 0.35), even when controlling for response time at the item level—indicating a detrimental effect on cognitive processing beyond speeding. Third, time pressure disproportionately reduced response times for difficult items and participants with high ability, working memory capacity, or need for cognition, although this did not differentially affect ability estimates. Overall, both the review and empirical sections show that the effects of time pressure go well beyond forcing participants to speed or skip the last few items and make even mild time constraints inadvisable when attempting to measure maximal performance, especially for high-performing samples.

Tests of fluid intelligence (*Gf*) can be administered either untimed, or with a time constraint (usually at the test level, but sometimes as an item-level deadline: e.g., Kyllonen et al. 2018). Any investigator interested in measuring fluid intelligence has to decide between these two options. The choice is not an easy one, as it depends on how exactly measurement will be affected by time pressure.

Raven’s matrices, as the test most representative of fluid intelligence (Carpenter et al. 1990), are a good illustration of the dilemma. On one hand, the test was explicitly designed to be completed untimed. John C. Raven (1938) noted that the progressive matrices “cannot be given satisfactorily with a time-limit”; John Raven (2008) remarked that “it would not make sense to set a time limit within which people have to show how high they can jump whilst also insisting that that they start by jumping over the lowest bar. Clearly, the most able would not be able to demonstrate their prowess […] it also follows that it makes no sense to time the test”.

On the other hand, a long testing time is an obstacle in many situations: a few participants in my lab have prolonged a testing session for over an hour trying to solve every single item in Raven’s Advanced Progressive Matrices (APM), which is psychologically interesting but logistically troublesome. This quickly led investigators to experiment with time limits (e.g., Bolton 1955). Short forms were developed (Arthur and Day 1994; Bilker et al. 2012; Bors and Stokes 1998); various time limits were tested (Hamel and Schmittmann 2006), and norms were ultimately made available for different time limits (Raven et al. 1998). The end result is that as with most intelligence tests (Wilhelm and Schulze 2002), in contemporary assessment, Raven’s matrices are often administered with a time constraint.

Is imposing time pressure a good or a bad thing? Time pressure has a limited detrimental effect on discriminating power (a reasonable time limit still allows most participants to finish most items, save for the final and most difficult items, which tend to have low success rates anyway; e.g., Bolton 1955), on reliability (e.g., Bolton 1955; Poulton et al. 2022; see also Hong and Cheng 2019), and on the dimensional structure (Poulton et al. 2022) of Raven’s matrices. However, this limited impact on basic psychometric properties does not mean that versions with or without a time limit are equivalent (e.g., Davidson and Carroll 1945; Rindler 1979). A more important question is whether time pressure impacts the validity of the task.

Time pressure can constitute a major threat to validity (Lu and Sireci 2007); this point has been recognized for a long time (Cronbach 1949). A speeded version of Raven’s matrices tends to correlate very well with the same task performed without a time limit (Hamel and Schmittmann 2006), but this is not the only aspect of validity. Time pressure may affect the response processes which translate individual differences of reasoning ability into differences of performance (Borsboom et al. 2004; Borsboom and Mellenbergh 2007). In other words, if forcing participants to respond faster changes the way items are processed, in such a way that performance is less dependent on the reasoning processes the task is supposed to be measuring, then a time limit should not be used. A meta-analysis based on Raven’s matrices indicated that using a time limit substantially changes correlations between reasoning performance other constructs, suggesting that response processes are indeed affected by time pressure (Tatel et al. 2020).

The literature has extensively covered various aspects of the effect of a time pressure on response processes and validity in intelligence tasks (e.g., Kyllonen and Zu 2016). Six main potential effects of a time pressure (and potential threats on task validity) can be listed: (1) preventing completion of certain items, (2) involving an additional contribution of mental speed, (3) constraining response times on items, (4) modifying aspects of cognitive processing of the items, (5) affecting psycho-affective variables such as test anxiety and motivation, and (6) differentially affecting individuals as a function of individual abilities (e.g., working memory). These potential effects of time pressure overlap to an extent (e.g., constraining response times may force qualitative changes in item processing). The next sections provide a brief summary of these six potential effects, before listing the unanswered questions that provided the impetus for the current study.

## 1. Brief Literature Review of the Potential Effects of Time Pressure

### 1.1. Effect 1: Time Pressure Leads to Skipping Items

When performing an intelligence test under time pressure, some participants may lack enough time to finish the task. The task is then interrupted before completion, which means some items are never reached and never attempted by the participant, leading to a lower score. This means that a participant’s score no longer necessarily reflects their maximal level of reasoning performance (e.g., Goldhammer 2015), in the sense of the maximum number of problems they should have been able to solve given their level of intellectual ability (see also Raven 2008).

This effect of time pressure on the omission of some problems has been the most discussed by classic psychometrics. It constitutes the basis of statistics that aim to summarize the effects of speededness based on the amount of items not reached by participants (e.g., Cronbach and Warrington 1951; Gulliksen 1950b; Stafford 1971). A similar rationale is implicit in factor analyses estimating a speededness factor based on the last, but not the first items (Borter et al. 2020; Estrada et al. 2017), in factor analyses assigning a loading on the speededness factor that increases with item serial position (e.g., Schweizer and Ren 2013), in attempts to estimate processing speed based on the number of omitted items (e.g., Schweizer et al. 2019a), and in the finding of poorer model fit for later items (Oshima 1994).

One major challenge with the omission of certain items is that it could interact with test-taking strategies. Indeed, some participants may deliberately decide to spend enough time on early problems, with the risk of running out of time and having to skip later items, whereas others may prefer to proceed quickly throughout the whole test (Goldhammer 2015; Semmes et al. 2011). These test-taking strategies may possibly interact with individual differences, with more able participants being more skilled at managing their time and selectively speeding up or slowing down depending on item difficulty and remaining time (van der Linden 2009). It is also noteworthy that some participants may choose to keep a margin of security, leading them not to use all the time they have available and finish a test or item before the deadline (see Bolsinova and Tijmstra 2015). Conversely, there may be individual catch-up phenomena, so that participants speed on early items but selectively slow down later when they have time left on the counter.

### 1.2. Effect 2: Time Pressure Taps into a Speed Factor

Intelligence tests administered with a speed constraint tend to yield results that correlate well with an untimed version of the same task (Preckel et al. 2011; Vernon et al. 1985; Wilhelm and Schulze 2002), which suggests that despite shifting the focus from a pure power test to a mix of power and speed (Gulliksen 1950a), speededness does not radically alter the nature of the task. However, speeded intelligence tests tend to give rise to a speed factor in factor analysis (Ren et al. 2018; see also Estrada et al. 2017; Schweizer and Ren 2013), and there are indications that scores on a speeded reasoning test are a composite of unspeeded reasoning and processing speed (Wilhelm and Schulze 2002). Conversely, taking into account participant speed can improve model fit in confirmatory factor analysis of speeded reasoning tasks (Schweizer and Ren 2013; Schweizer et al. 2019a, 2019b; see also Semmes et al. 2011; Wollack et al. 2003). More generally, speeded reasoning tasks tend to correlate better with other speeded than unspeeded measures (Wilhelm and Schulze 2002). These results all suggest that imposing a time limit in a matrix task invokes an additional contribution of mental speed.

Some theorists may consider the involvement of mental speed as a good thing. Many studies have shown a substantial correlation between tests of mental speed and performance on reasoning tests (both speeded and unspeeded: Vernon et al. 1985; Vernon and Kantor 1986). For this reason, mental speed may be viewed as an instrumental ability that supports the operation of intelligence: faster participants may, for example, be better able to maintain information relevant to logical reasoning in working memory before it decays. Along those lines, mental speed has long been investigated as a possible contributor to individual differences in reasoning performance (e.g., Ackerman et al. 2002; Conway et al. 2002; Vernon 1983), as well as a contributor to the development of intelligence in childhood (Coyle 2013; Demetriou et al. 2013; Fry and Hale 1996, 2000; Kail and Salthouse 1994; Kail 2000, 2007) and its decrease in aging (Babcock 1994; Salthouse 1992, 1996).

Alternatively, some authors view processing speed as a fundamental component of intelligence (e.g., Vernon 1983): Jensen in particular speculated that processing speed could reflect basic differences at the neurological level, which could constitute a major underpinning of the general factor *g* (Jensen 1993, 1998). A related argument comes from the factor structure of intelligence: the Cattell–Horn–Carroll (CHC) theory of cognitive abilities explicitly includes speed factors as broad abilities under the general factor (McGrew 2009; Schneider and McGrew 2018; see also McGrew 2023). This view makes mental speed an integral part of intelligence as a construct, and if mental speed is part of what we mean by “intelligence”, then forcing participants to work quickly should just tap into an additional dimension of intelligence, leaving task validity unaltered or even enhanced.

This argument has multiple problems, however. First, the observed correlation between mental speed and intelligence does not necessarily imply an important causal status for mental speed (e.g., Schubert et al. 2018), and it is doubtful whether mental speed actually has real-life implications that make it worth measuring (Kyllonen and Zu 2016). Second, imposing a time limit and contaminating an intelligence test with speed-related variance can spuriously inflate correlations with other constructs also measured under time constraints (e.g., Ackerman et al. 2002; Engle and Kane 2004; Tatel et al. 2020). Third, although cognitive psychology often presents “mental speed” as a unitary ability, it is in fact a complex multidimensional construct (see Danthiir et al. 2005; Roberts and Stankov 1999; see also Draheim et al. 2019, for a discussion of measurement issues). As a result, the CHC theory comprises multiple factors related to speed: processing speed in simple cognitive tasks (*Gs*), reaction and decision speed for elementary single items (*Gt*), speed in motor activities (*Gps)*, and rate and fluency for retrieval of information stored in long-term memory (*Gr*). The relation between these factors (e.g., do they form a superordinate speed factor?) is currently unclear (Schneider and McGrew 2018). Moreover, the speed at which a complex reasoning task can be performed does not map cleanly on any CHC factor and probably taps into a mix of *Gf* and one or more of speed factors (including Gs, but also Gt in certain tasks, and possibly Gr which encompasses ideational fluency; see Schneider and McGrew 2018). Fourth, speed is not solely a question of ability and also depends on motivation, personality, and an individual’s speed-accuracy tradeoff (Shaw et al. 2020). Lastly, it is not even certain that the speed factor that appears under time constraints actually represents mental speed: in some cases, it may also reflect individual ability and individual strategies to deal with the time pressure (Davison et al. 2012; Semmes et al. 2011) or a different construct altogether such as a form of rule generation fluency (Verguts et al. 1999). In short, imposing a time limit to a reasoning task and convoking a speed factor make the measure less tractable overall.

### 1.3. Effect 3: Time Pressure Constrains Response Times

Time pressure naturally encourages speeding in the task and therefore constrains the amount of time that can be spent on a given item. This may be viewed as a threat for validity or not, depending on whether a high speed of responding is taken as a reflection of high intelligence. As noted by Schneider and McGrew (2018), “the speed metaphor is often used in synonyms for *smart* (e.g., *quick-witted*)”. In this view, it is inherently desirable to solve intellectual problems more quickly: if two participants have the same accuracy, it makes intuitive sense to believe that the faster one is more intelligent (Thorndike et al. 1926). This approach considers speed as an integral aspect of performance in the task. One way to take this into account is to use composite scores that combine accuracy and speed (e.g., Bruyer and Brysbaert 2011; Dennis and Evans 1996; another example is found in certain subtests of Wechsler scales, which give bonus points for quick answers) or to jointly model accuracy and response times (Goldhammer and Kroehne 2014; Klein Entink et al. 2009b).

With this perspective, the speed at which the response process is executed is an index of its effectiveness as much as the correctness of the response. Therefore, imposing a time limit and constraining time on task is not necessarily a problem (if the difficulties posed by problem complexity and limited time both challenge the same ability, then high-performing participants should be both faster and more accurate) and could even be viewed as an advantage (since a time limit constrains the response times of participants, this could make them more comparable in terms of accuracy: see Goldhammer 2015; see also Bolsinova and Tijmstra 2015).

However, this line of reasoning overlooks a critical aspect of solving complex intelligence tests: being fast is not necessarily a good thing. There are at least two ways to frame this idea. The first is to stress the fact that cognitive operations take time: limiting the amount of available time mechanically limits the number of operations that can be completed. Given that complex operations germane to fluid reasoning (such as rule induction) are constrained by simpler operations related to basic manipulation of information, time pressure is likely to affect complex operations to a greater extent (Salthouse 1996). The other important point is that speed is not only an index of effective reasoning: a low speed also reflects carefulness (Kyllonen and Zu 2016). In terms of cognitive processes, longer response times can largely reflect time spent for validation and evaluation of one’s response (Goldhammer and Klein Entink 2011); one study showed that participants who care more about the results tend to respond more slowly (Klein Entink et al. 2009a).

Empirical data have substantiated the idea that responding slowly can be positive. At the item level, an unpublished study of 159 participants with eye-tracking showed that longer fixations on a matrix problem were associated with better performance, which suggests that taking the time for reflection is beneficial (de Winter et al. 2021). At the task level, RTs tend to be positively correlated with ability estimates, which means better participants tend to be slower (DiTrapani et al. 2016; Goldhammer and Klein Entink 2011; Klein Entink et al. 2009b; Partchev and De Boeck 2012). In the case when participants give fast responses, speed is especially negatively correlated with success rate (Partchev and De Boeck 2012; note that this result was specific to Raven’s matrices and did not occur for a verbal analogies task).

Critically, the emphasis on slow responding appears to depend on ability and difficulty (Goldhammer et al. 2014). Participants with a higher level of ability and/or motivation tend to modulate their RTs as a function of problem difficulty and spend much longer on difficult problems (Perret and Dauvier 2018; Gonthier and Roulin 2020; see also Tancoš et al. 2023), suggesting that these require substantially more time to be solved correctly. In line with this view, the relation between RTs and accuracy is negative for easy problems but becomes less negative (Dodonova and Dodonov 2013) or even positive for more difficult problems (Becker et al. 2016; Goldhammer et al. 2015). In terms of processing, it is likely that complex problems, which involve more logical rules and more components on which to apply these rules, require more time to elaborate a correct answer. In short, responding slowly can also be characteristic of high performance, especially for difficult problems and high-ability participants. It is also worth recalling that not all groups respond at the same speed: forcing fast responses may be more detrimental to participants with a slower response speed, such as young children (Borter et al. 2020) and older adults (Salthouse 1996).

### 1.4. Effect 4: Time Pressure Can Affect Cognitive Processing

Encouraging speeding when responding to a problem may conceivably affect cognitive processing, above and beyond limiting the amount of processing that can be performed. A few studies have even suggested that fast responses to an intelligence test involve a different ability or process than slow responses (Partchev and De Boeck 2012; DiTrapani et al. 2016), although no information was provided regarding the nature of this ability. There are multiple pathways by which cognitive processing could be affected.

At the item level, one possible way to conceptualize the possible effects of time pressure is to think of the response process in a reasoning task as a drift-diffusion model (e.g., Frischkorn and Schubert 2018; Kang et al. 2022; Lerche et al. 2020; van der Maas et al. 2011). This class of models considers that when confronted with a problem, participants continuously accumulate evidence in a random walk process (modeled as a constant drift rate in the direction of the response, plus noise), until they reach a decision threshold. Encouraging participants to speed their responding due to a time limit could force them to lower their decision threshold, interfering with verification of their response as discussed in the previous section (Goldhammer and Klein Entink 2011; Klein Entink et al. 2009a; Kyllonen and Zu 2016). This would translate as faster RTs, lower accuracy, and lower confidence in one’s response.

Apart from a change of decision threshold, time pressure could also force participants to accumulate information at a higher rate. Based on the decision-making literature, this could translate into several effects in terms of cognitive processing (Johnson et al. 1993; see also Ben Zur and Breznitz 1981; Wright 1974), including acceleration (performing the same cognitive operations more quickly), filtration of information (considering less information before making a decision; see also Salthouse 1996), or a change of strategy (tackling the task in a qualitatively different way). Acceleration or filtration would translate as faster responses in the task and lower accuracy; filtration in particular could also translate as lower accuracy conditional on RT, i.e., lower accuracy for the same RT, owing to the qualitatively different nature of information processing.

As for changes of strategy, there has been little study of the effects of time pressure on strategy use in intelligence tests, but such effects seem especially likely. Participants in complex learning tasks tend to switch to faster or more simple strategies under time pressure (see Chuderski 2016); the same phenomenon is observed in mathematics tasks (Caviola et al. 2017) and is assumed to occur in working memory tasks (Friedman and Miyake 2004; Lépine et al. 2005; St Clair-Thompson 2007; Thomassin et al. 2015). In the context of a matrix task, a change of strategy could mean turning away from the effective constructive matching strategy (Chuderski 2016), which relies on the time-intensive process of reconstructing the correct answer by integrating all information in an item, to the less costly strategy of response elimination, which relies on testing each possible answer in turn to see if it seems to superficially fit the matrix (for a review, see Laurence and Macedo 2022; see also Bethell-Fox et al. 1984; Snow 1980). There is also substantial evidence that participants often adopt a strategy of rapid guessing when under severe time constraints (Attali 2005; Jin et al. 2023; Schnipke and Scrams 1997; Schweizer et al. 2021), which would mean turning away from both constructive matching and response elimination. Critically, rapid guessing may not be constant across groups and across individuals (e.g., Must and Must 2013), providing another source of potential individual differences.

The effects of time pressure on cognitive processing of a given item may also go beyond what can be modeled at the item level: time pressure could also be expected to negatively affect learning, disrupting performance in a cumulative fashion over the course of the task. Learning is an important aspect of performance in Raven’s matrices: participants discover logical rules over simple items and then generalize them over more complex items presented later in the test (Ren et al. 2014; Verguts and De Boeck 2002), either explicitly or as a form of implicit or associative learning (Ren et al. 2014). One study has suggested that time pressure is detrimental to learning in a matrix task (Chuderski 2016), possibly because giving faster responses on early items means participants process logical rules more superficially, in a way that impedes transfer to more difficult items. This mechanism could contribute to selectively increasing the detrimental effect of time pressure on items presented towards the end of a test, although the particular design of this study (with participants completing two samples of items in the task in succession, without then with time pressure) makes it difficult to know if this effect would occur under more classic testing conditions.

### 1.5. Effect 5: Time Pressure Can Affect Anxiety and Motivation

Apart from direct effects due to the time restriction, it is also possible that the pressure itself has an effect on accuracy. Studies from the decision-making literature have suggested that participants perform worse under a time pressure, not only when there is an actual time restriction (Cella et al. 2007) but also when there is a *perceived* time pressure, even in the absence of any time manipulation (DeDonno and Demaree 2008).

This phenomenon could be partly due to an effect of pressure on constructs related to intelligence: for instance, time pressure could decrease participant motivation to complete the task. One study showed that participants who had to complete a reasoning task under an explicit time pressure were less intrinsically motivated, as reflected in both lower ratings of interest and less time spent voluntarily engaging with the task materials after the end of the testing session (Amabile et al. 1976). Under this view, time pressure could also conceivably change the relation between performance and motivation (see Kuhn and Ranger 2015).

Perceived time pressure could also create stress or test anxiety in participants (e.g., Sussman and Sekuler 2022). This could interfere with performance in several ways, such as creating worrisome thoughts which use up resources in working memory (Eysenck and Calvo 1992; for other examples, see Ashcraft and Kirk 2001; Moran 2016), although this mechanism is disputed (Kellogg et al. 1999). This process has been mostly studied in the related contexts of academic achievement and math anxiety (Caviola et al. 2017) and may also occur with intelligence tests. Time pressure could also conceivably interact with individual differences in anxiety: in the case of math reasoning, removing time pressure is sometimes observed to selectively increase performance for more anxious participants (Plass and Hill 1986), although this is not always the case (Kellogg et al. 1999; see also Traub and Hambleton 1972).

### 1.6. Effect 6: Differential Effects of Time Pressure

Although time pressure does not seem to affect the relative position (rank-ordering) of participants to a large extent (Preckel et al. 2011; Vernon et al. 1985; Wilhelm and Schulze 2002), time pressure could still be expected to interact with individual differences in ability in absolute terms so that the distance between high-ability and low-ability participants varies as a function of time pressure. A situation often observed in reasoning tasks is the choking under pressure effect, wherein imposing a pressure (such as instructions emphasizing the measurement of intelligence, the addition of social pressure, dual tasking, etc.) creates a larger decrement of performance for high-performing participants, especially those with high working memory capacity (WMC; Gimmig et al. 2006; for examples with math tests, see Beilock and Carr 2005; Beilock and DeCaro 2007). Choking under pressure could also occur with time pressure, decreasing the distance between low- and high-ability participants.

The same effect could occur with WMC, instead of ability: time pressure has been observed to decrease the distance between low- and high WMC participants (Colom et al. 2015), which could be problematic given that WMC is one of the major correlates of intelligence. On the other hand, the opposite effect has also been reported: it has been argued that speeded intelligence tests have higher correlations with WMC (Chuderski 2013, 2015; Tatel et al. 2020) because time pressure requires participants to integrate all information in working memory, leaving no time to decompose the problem. This would lead to time pressure *increasing* the distance between low- and high-ability participants. This finding however was not replicated in other studies (Colom et al. 2015; see also Ren et al. 2018).

Apart from WMC, there is suggestive evidence that time pressure could increase the relation between performance in Raven’s matrices and spatial abilities (Tatel et al. 2020). A differential effect of time pressure could also conceivably be found with other constructs, such as motivation: given that more motivated participants tend to spend longer on problems (e.g., Wise and Kong 2005), imposing a time pressure could selectively decrease the performance of participants with high motivation. Lastly, a differential effect could be found as a function of mental speed and more generally as a function of age: time pressure could disproportionately affect younger children with low mental speed (Borter et al. 2020) and possibly older adults although this is not necessarily the case in practice (Babcock 1994).

Given the fact that high-ability participants tend to modulate their RTs to spend selectively more time on more difficult items (Gonthier and Roulin 2020; Perret and Dauvier 2018; Tancoš et al. 2023), all these possible differential effects might also be expected to interact with item difficulty: if time pressure affects high-ability participants to a larger extent, it may be even more true for the most difficult items. However, RT modulation in the face of difficulty is a relatively new topic in the literature, and this possibility has not been tested.

### 1.7. Unanswered Questions and Rationale for the Experimental Study

As reviewed in the preceding sections, there are many potential effects of time pressure on response processes in an intelligence test. Most of these expected effects have the potential to be detrimental to task validity: forcing some participants to skip some items depending on their test-taking strategy, convoking an intractable speed factor, restricting RTs selectively for high difficulty items and high-ability participants, encouraging filtration of information or guessing strategies, decreasing motivation and increasing anxiety, and decreasing the distance between high- and low-ability participants or strengthening the correlation with other constructs would not be desirable when attempting to estimate intellectual ability.

Although some of these effects have been largely studied (especially Effect 2: the emergence of a speed factor under speeded testing), many remain largely speculative in the specific context of intelligence tests. Covering all these topics would be difficult for a single study, but a few analyses can provide tentative answers to many of them. The empirical section of this work was designed to cover three broad unknowns in the intelligence testing literature.

The first is the actual extent of speeding in an intelligence test performed under time pressure. It is clear that on average, participants respond more quickly under time pressure (see Effect 3: time pressure constrains response times). It is less clear to what extent this speeding affects all participants (is there a shift in the whole distribution of RTs, or is the average lower because of just a few participants who respond more quickly?) and all items (are just the final items affected due to lack of available time towards the end of the test, or do participants speed up for all items?). This question is closely related to the way participants manage their time on task (see Effect 1: time pressure leads to skipping items). Do participants use up all their available time; do they finish with a margin of security as advocated by some, or do they run out of time and omit the final items as proposed by others? To what extent do test-taking strategies regarding omissions vary across participants? Are there catch-up phenomena such that participants slow down or accelerate throughout the task under time pressure, ultimately catching up with participants under different conditions of time pressure?

The second question is the mechanisms by which time pressure can be detrimental to performance. Is it just a question of participants failing to complete the final items due to insufficient time (see Effect 1: time pressure leads to skipping items)? Does time pressure induce speeding that restricts the number of cognitive operations that can be performed, leading to lower accuracy (see Effect 3: time pressure constrains response times)? Or does time pressure have a broader impact on cognitive processing, above and beyond speeding, e.g., in terms of filtration of information, responding with a lower confidence threshold, using less effective strategies (see Effect 4: time pressure can affect cognitive processing), or conative aspects of the task (see Effect 5: time pressure can affect anxiety and motivation)? Would time pressure still have a detrimental effect on accuracy when controlling for response time on a given item?

The third question is the way time pressure affects participants as a function of individual differences (see Effect 6: differential effects of time pressure). Is it the case that time pressure selectively increases the effect of individual differences in ability, working memory, or motivation, as predicted by some authors, or decreases their effect, as predicted from the hard fall effect framework? Does time pressure affect individual differences in relative terms (rank-ordering of participants) or in absolute terms (score difference between participants)? How does time pressure affect individual differences at the item level, including the selective modulation of RTs by high-ability participants on difficult items (see Effect 3: time pressure constrains response times)?

To answer these questions and better understand the effects of time pressure on response processes in an intelligence test, different conditions of time restriction were compared in a matrix reasoning task. The task was Raven’s APM (abridged to 18 items), chosen both because it is widely used and because it is the task with the most information available regarding response processes and their relation to time. Three conditions of time restriction were used: unrestricted time (with no instructions regarding time or response speed), 20 min, and 10 min. The 20 min and 10 min time limits were selected based on a prior study without a time limit (Gonthier and Roulin 2020): 20 min were sufficient for virtually all participants to complete the 18 items of the task, whereas 10 min were sufficient for less than half the participants to complete the task. The 20 min condition matches the time usually allowed for Raven’s matrices (40 min for the full 36 items), whereas the 10 min condition is in the range of studies using highly speeded versions (e.g., Babcock 1994; Hamel and Schmittmann 2006; Ren et al. 2018; Unsworth et al. 2009).

For each item, the task recorded accuracy, response time, confidence of the participant in their answer, and use of the constructive matching and response elimination strategies. Individual differences were also assessed for two constructs related to performance in the task: working memory, as a window into the relation between performance and cognitive ability, and need for cognition (NFC: the tendency to engage in and enjoy complex thinking, reflecting intrinsic motivation to solve reasoning problems; see Gonthier and Roulin 2020), as a window into the relation between performance and motivation as a function of time pressure. The effect of time pressure on accuracy, RT, confidence, and strategy use was assessed both at the task level and at the item level, with additional analyses testing relations with working memory and need for cognition as a function of time pressure.

The data were analyzed to answer the three broad questions listed above. The first question concerning the extent of speeding was tested by analyzing time on task, the distribution of item omissions, average RTs at the task level, and RTs at the item level, including RT distributions. The second question concerning the mechanisms by which time pressure can affect accuracy was tested by analyzing accuracy, confidence, and strategy use at the task and item level (the effect of time pressure on anxiety and motivation was not tested in this study) and by modeling accuracy conditional on RTs. The third question concerning individual differences was tested by analyzing the linear and nonlinear effects of ability, working memory and NFC on accuracy, and RTs at the task level, as well as their effects on modulation of RTs at the item level.

## 2. Method

### 2.1. Participants

A sample of 300 undergraduate students at the University of Rennes 2 participated for course credit. Five participants were removed due to failing to complete the working memory task (failing to reach the criterion of minimal processing accuracy; see Unsworth et al. 2005), leaving a total sample of *N* = 295. Participants were randomly assigned to one of the three experimental conditions: untimed (*n* = 97, 80 females and 17 males; mean age = 19.33 years, *SD* = 1.71), 20 min (*n* = 99, 77 females and 22 males; mean age = 20.04 years, *SD* = 3.26), or 10 min time pressure (*n* = 99, 86 females and 13 males; mean age = 19.55 years, *SD* = 3.57). All participants were native French speakers, and none had completed any of the experimental tasks before. All participants provided written informed consent prior to the experiment.

### 2.2. Materials

#### 2.2.1. Raven’s Advanced Progressive Matrices

Participants completed Set II of Raven’s APM (Raven et al. 1998). Each item is composed of a 3 × 3 matrix of black-and-white figures, where the bottom right piece is missing; participants are required to select the figure that logically completes the matrix, among eight possible answers. Each participant completed only odd-numbered items, leaving 18 of the 36 items, as in prior studies (e.g., Gonthier et al. 2016; Jastrzębski et al. 2018; Unsworth et al. 2010).

After each APM problem, participants were required to answer two questions about the strategies they used (based on Gonthier and Roulin 2020; see also Gonthier and Thomassin 2015): one assessing constructive matching (*After examining the drawing, you imagined the missing piece before looking for it among the possible answers*) and one assessing response elimination (*You examined each possible answer in turn to decide whether it could be the missing piece*). The two questions were presented on the same screen; participants were asked to rate their agreement with each proposition on a 9-point Likert scale. This also served to compute a composite score representing effective strategy use (as constructive matching—response elimination). On the next screen, participants were asked to rate their confidence in the fact that their answer to the APM problem was correct, on a visual analogue scale ranging from 0% to 100% (see Mitchum and Kelley 2010).

The time pressure manipulation was implemented as follows. Participants were instructed that they would have to solve 18 problems in ascending order of difficulty; in the 20 min and 10 min conditions, the following sentence was added: *WARNING: You only have 20/10 min to solve these problems*. During the task, a counter appearing in the top left corner of the screen displayed item progression (e.g., *1/18*) for all participants and remaining time (e.g., *09’ 58”*) for participants in the 20 min and 10 min condition. This counter was displayed only along with matrices and was hidden for the strategy and confidence rating questions; participants were instructed that time was only deducted when working on a matrix problem. Due to the presence of the confidence and strategy use rating questions, participants were not allowed to backtrack to a previously answered problem.

#### 2.2.2. Working Memory Capacity

Working memory capacity was measured with the Composite Complex Span (CCS), which has satisfying reliability and convergent validity with the APM in student samples (Gonthier et al. 2016). The CCS is a French-speaking adaptation of three complex spans (see Conway et al. 2005; Redick et al. 2012), where participants have to alternate between solving simple problems and memorizing unrelated stimuli. The tasks are the reading span (participants have to decide whether sentences are correct while memorizing digits), symmetry span (decide whether spatial displays are symmetrical while memorizing locations in a 4 × 4 grid), and operation span (deciding whether math operations are correct while memorizing consonants; see Unsworth et al. 2005). At the end of a trial, all to-be-memorized stimuli have to be recalled in serial order. The CCS includes a total of 22 trials, with set sizes ranging from 3 to 8.

Performance in a trial was computed using the edit-distance scoring method, an improved variant of partial-credit scoring with better psychometric properties (see Gonthier 2022). With edit-distance scoring, the score for a trial is equal to the set size minus the number of changes required to edit the participant’s response into the correct sequence (e.g., for the target ABCDE, recalling BADE means two changes are required—inverting the position of A and B and adding a C—which nets a score of 3 out of 5). Performance was summed across all trials in a complex span; then, the three complex span scores were standardized and averaged to yield a domain-general WMC estimate^1^.

#### 2.2.3. Need for Cognition

Need for cognition was assessed with a French-speaking adaptation (Salama-Younes 2011) of the 18-item short form of the need for cognition scale (Cacioppo et al. 1984). Participants rated their agreement with 11 propositions (such as *I prefer simple problems to complex problems*) on a 4-point Likert scale.

### 2.3. Procedure

Participants performed the testing session in groups of 2 to 12 individuals in a university computer room. The first task of the experimental session was the CCS, which lasted approximately 25 min. After a short break, participants completed two training items from Set I of the APM, followed by the rest of the APM task. The whole experimental session lasted approximately 40 to 60 min.

### 2.4. Data Analysis

Reliability was estimated based on internal consistency using Cronbach’s alpha coefficients; these coefficients were compared across conditions using the Feldt test (Feldt 1969; computed using package cocron for R: Diedenhofen and Musch 2015; R Core Team 2023). The effect of time pressure on average performance was analyzed using analyses of variance (ANOVAs), followed by post hoc comparisons between the three conditions using Tukey’s HSD correction.

The role of individual differences at the task level was primarily tested using general additive models (GAMs; see Wood 2017), which are similar to linear regressions, extended to include non-linear effects of predictors. Statistical tests in GAM analyses are reported based on approximate *p*-values (see Wood 2017), along with effective degrees of freedom (*edf*: effective degrees of freedom equal to 1 reflect a linear relationship between predictor and dependent variable; values greater than 1 reflect a more complex trajectory).

Data at the item level were analyzed using general additive mixed models (GAMMs), which include random effects for each participant, allowing for data analysis at the item level (see Gonthier and Roulin 2020; Perret and Dauvier 2018). GAM and GAMM analyses were performed using the *mgcv* package (Wood 2017; version 1.8-42) for *R* (R Core Team; version 4.2.1). All dependent variables were modeled assuming a gaussian distribution (inference based on *F*-tests), except for accuracy at the item level, which used a binomial distribution (inference based on χ^2^); log-transforming RTs did not change the pattern of results, so the data are presented without transformation to make interpretation easier. Subject-level random effects were modeled as random intercepts; models were fit using restricted maximum likelihood; basis dimension was adjusted so as to be sufficient for all analyses; smooths were modeled with the default classes (see also Gonthier and Roulin 2020).

## 3. Results

The data for this experiment and sample R code are available at https://osf.io/9rtxf/ (uploaded 12 June 2023).

A preliminary analysis showed that sample composition was equivalent in the three conditions: there were no significant differences between conditions in terms of sex: χ^2^(2) = 2.82, *p* = 0.244; age: *F*(2, 287) = 1.47, *p* = 0.231, η^2^_p_ = 0.01; WMC: *F*(2, 287) = 0.02, *p* = 0.980, η^2^_p_ = 0.00; or NFC: *F*(2, 287) = 0.20, *p* = 0.822, η^2^_p_ = 0.00.

Descriptive statistics for the APM at the task level are available in Table 1. Internal consistency was acceptable overall, especially in the Unlimited time and 20 min conditions. There was a clear pattern of decreasing reliability with a high time pressure in the 10 min condition for both accuracy and RTs, with reliability below the conventional threshold of .70 for both measures. The difference between conditions was significant for RTs, χ^2^(2) = 15.37, *p* < 0.001, but not accuracy, χ^2^(2) = 2.38, *p* = 0.304. Reliability was high all around and unaffected by time pressure for constructive matching, χ^2^(2) = 1.80, *p* = 0.406, and response elimination, χ^2^(2) = 1.41, *p* = 0.495. For confidence ratings, there was a significant effect of time pressure, χ^2^(2) = 8.53, *p* = 0.014, reflecting higher reliability in the 20 min condition and no difference between the Unlimited time and 10 min condition, but reliability was high all around.

### 3.1. Time on Task and Missed Items

Participants in the Unlimited time condition spent on average 11.94 min on the task (*SD* = 4.63, range = 3.96–30.37 min). Percentile ranks for time-on-task in this condition, along with the corresponding item completion rate, are given in Table 2. Overall, the fastest participants needed approximately 6 min to complete the 18 items of the task; the median participant needed approximately 11 min, and almost all participants were finished by 20 min. In other words, the median completion rate on the APM was about 1.5 items per minute, and most participants comfortably solved about 1 item per minute.

By contrast, participants in the 20 min condition spent on average 10.13 min on the task (*SD* = 3.56, range = 3.17–20 min), and participants in the 10 min condition spent on average 7.99 min on the task (*SD* = 1.56, range = 3.19–10 min). In other words, participants spent less time on the task on average under time pressure and had a tendency not to use all of the allotted time: even with the severe pressure of the 10 min condition, the average participant finished with about 20% of time left.

**Figure 1:**
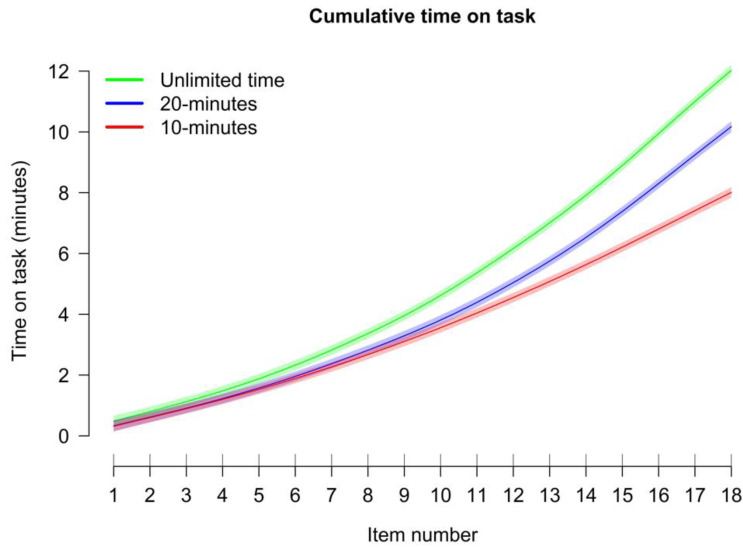
Cumulative time on task as a function of condition. Confidence bands represent +/−1 standard error.

Cumulative times on task as a function of item position were analyzed with a GAMM. The results, as represented in Figure 1, showed that there was no catch-up phenomenon (such as participants pausing or slowing down at some point, decreasing the distance between conditions): under time pressure, participants generally proceeded through the task more quickly and reached every item sooner on average, and the difference between conditions increased in a cumulative fashion throughout the task.

An analysis of items missed due to elapsed time showed that the 20 min condition had four missed items for 99 participants (0.22% of the total), three of which were missed by the same participant. By contrast, the 10 min condition had 40 missed items for 99 participants (2.24% of the total), broken down as follows: 0 missed item (*n* = 84 participants), 1 missed item (*n* = 5), 2 missed items (*n* = 6), 3 missed items (*n* = 2), 7 missed items (*n* = 1), and 10 missed items (*n* = 1). In other words, the dominant pattern under severe time pressure was for a participant to attempt all items, but a small minority of participants spent more time on early items and never reached more difficult items in the task. Altogether, it is clear that the total amount of missed items was small enough that detrimental effects of time pressure, as discussed in the next section, could not be attributed to omissions.

### 3.2. Effects of Time Pressure at the Task Level

**Figure 2:**
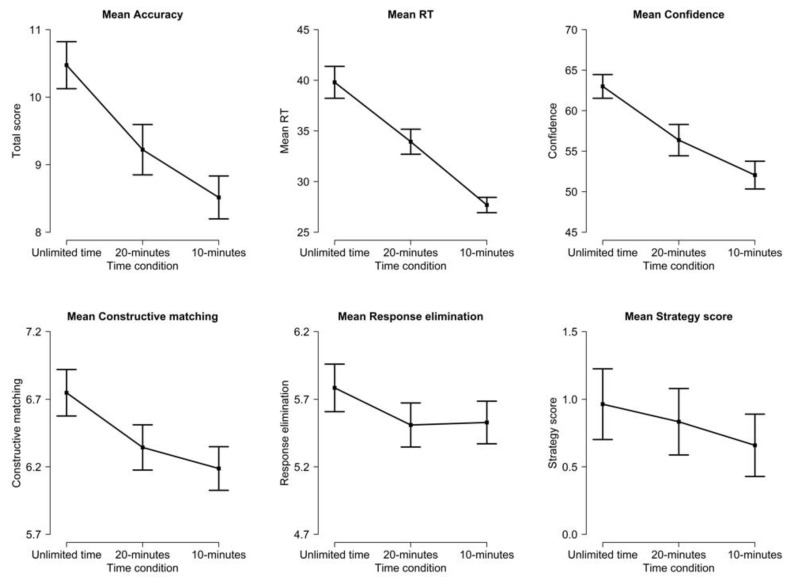
Effect of time pressure on total accuracy, mean RT (mean seconds per item), mean confidence (0 to 100%), mean constructive matching (1 to 9), mean response elimination (1 to 9), and mean strategy score (constructive matching—response elimination). Error bars represent +/−1 standard error of the mean.

All variables had distributions close to normal, except for the expected slight positive skewness for RTs. ANOVAs for the effect of time pressure at the task level are summarized in Table 3 and represented in Figure 2. The results showed significant effects of time pressure on accuracy, RTs, and confidence: a higher time pressure was associated with lower accuracy, faster RTs, and lower confidence in one’s response. There were moderate to large effect sizes for the pairwise comparisons between the Unlimited and 20 min conditions (Cohen’s *d* = 0.35 for accuracy, 0.42 for RTs, 0.39 for confidence), between the 20 min and 10 min conditions (*d* = 0.21 for accuracy, 0.61 for RTs, 0.24 for confidence), and of course between the Unlimited and 10 min conditions (*d* = 0.59 for accuracy, 1.00 for RTs, 0.69 for confidence).

Follow-up comparisons with Tukey’s correction showed that for both accuracy and confidence, only the difference between the Unlimited time condition and the 20 min time condition reached significance. In other words, accuracy and confidence decreased due to time pressure, even when this time pressure was sufficient for the majority of participants to comfortably solve the task: there was comparatively less difference between the 20 min and 10 min condition, although both accuracy and confidence descriptively decreased further when time pressure increased (see Figure 2).

For strategy use, the results showed descriptively a decrease in both constructive matching and response elimination with increasing time pressure (see Figure 2). For constructive matching, the effect of time pressure was significant, with follow-up comparisons showing a significant difference between the Unlimited time condition and the 10 min condition. The effect of time pressure did not reach significance for response elimination or the strategy use score. Overall, these results are not compatible with participants turning from constructive matching to response elimination under time pressure: with a descriptive decrease in the reported use of both strategies, time pressure seemed to mostly induce an increase in random guessing.

An alternative way to analyze confidence ratings is to compute calibration (the correlation between a participant’s confidence and their accuracy across all items), which reflects the effectiveness of metacognitive monitoring of one’s performance. Average calibration was in the 0.40–0.50 range and significantly above zero (*p* < .001), and there was no effect of time pressure on calibration estimates (for the Unlimited time condition: *M* = 0.47, *SD* = 0.25; 20 min condition: *M* = 0.44, *SD* = 0.24; 10 min condition: *M* = 0.43, *SD* = 0.24), *F*(2, 292) = 0.67, *p* = 0.511, η^2^_p_ = 0.00, BF_01_ = 14.72. In other words, participants were capable of judging their own performance, and participants under time pressure were aware of their lower accuracy.

### 3.3. Effects of Time Pressure at the Item Level

**Figure 3:**
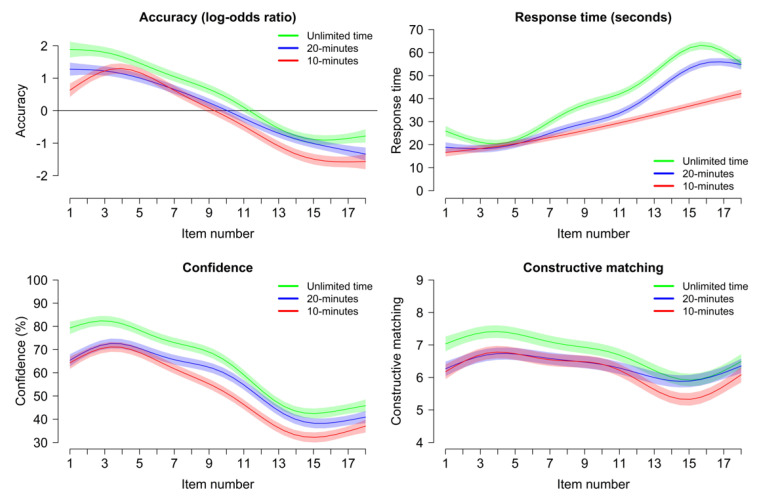
Effect of time pressure on accuracy (expressed as the log-odds ratio of a correct answer: 0 indicates a 50% chance of a correct answer), RT, confidence, and constructive matching, at the item level. Confidence bands represent +/−1 standard error.

For the four variables showing a significant effect of time pressure at the task level, data at the item level were analyzed with GAMMs (including the main effect of item position, as a function of time pressure). The results are displayed in Figure 3.

For accuracy, confidence in one’s answer, and constructive matching, there was a progressive decrease throughout the task as items became more difficult; this decrease was significant for all measures in all experimental conditions (all *p*s < .001). For accuracy, although the effect of time pressure was significant on average (as detailed in the previous section), there was no significant interaction between experimental condition and item position (all *p*s > 0.238). In other words, the effect of time pressure was homogeneous across all items: increasing time pressure was detrimental to accuracy, for all items to the same extent (see Figure 3). For confidence and constructive matching, there was a small difference in the 20 min condition, with less decrease throughout the task than for the Unlimited time and 10 min conditions (all *p*s < 0.046) and no difference between the Unlimited and 10 min conditions, but the difference was descriptively small (see Figure 3).

For RTs, there was a progressive increase throughout the task as items became more difficult, consistent with prior studies (e.g., Gonthier and Roulin 2020; Perret and Dauvier 2018); this was true in all three conditions (Unlimited time: *F* = 120.44, *edf* = 7.81, *p* < 0.001; 20-min: *F* = 101.48, *edf* = 7.58, *p* < 0.001; 10-min: *F* = 78.41, *edf* = 2.50, *p* < 0.001). This modulation of RTs as a function of item difficulty however depended on time pressure. In the Unlimited time condition, RTs increased to a large extent with item position, except for a drop for the last items, which appears to reflect participant disengagement in the face of difficulty (Gonthier and Roulin 2020). This modulation was significantly less extensive in the 20 min condition (*F* = 3.24, *p* = 0.008) and even less in the 10 min condition (difference between Unlimited time and 10 min conditions: *F* = 18.08, *p* < 0.001; difference between 20 min and 10 min conditions: *F* = 18.42, *p* < 0.001). In other words, time pressure substantially decreased the modulation of RTs as a function of item position, as reflected in a trajectory both closer to a straight line, and with a flatter slope (see Figure 3).

This conclusion was complemented by a more detailed analysis of RT distributions at the item level. For each separate item, mean RTs were compared using ANOVAs, with the results displayed in Table 4. Overall, there were significant effects of time pressure for 14 out of 18 items, confirming that speeding was not limited to the last few items. Participants in the 10 min condition answered significantly faster than participants in the Unlimited time condition in all cases. The 20 min condition fell between the two extremes: in the first half of the task, participants in this condition did not significantly differ from the other two or answered significantly faster than the Unlimited time condition; in the second half of the task, RTs in the 20 min condition were closer to the Unlimited condition and significantly slower than in the 10 min condition. Critically, the effect of time pressure was significant starting with the very first item of the task, confirming that speededness was partly the result of the time pressure itself rather than lack of available time.

**Figure 4:**
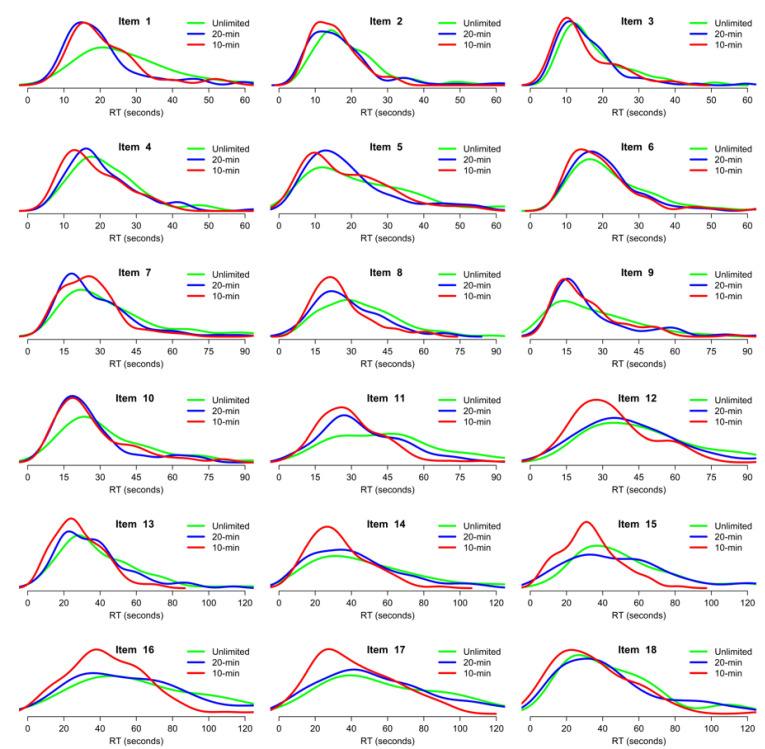
RT distribution for all items as a function of condition. The figure shows density estimates smoothed with a gaussian kernel.

Closer examination of RT distributions, as depicted in Figure 4, illustrated two additional points. First, speededness in the two conditions with time pressure was accompanied by a global shift in distributions, with reduced variance, for all items; in other words, the difference of average RT was not driven by a few participants who sped up under time pressure but by overall speeding for the whole sample. Second, RTs in the 20 min condition behaved inconsistently across items, with data indistinguishable from the 10 min condition in some cases (e.g., items 9–11) and indistinguishable from the Unlimited time condition in others (e.g., item 12).

### 3.4. Accuracy Conditional on Response Times

**Figure 5:**
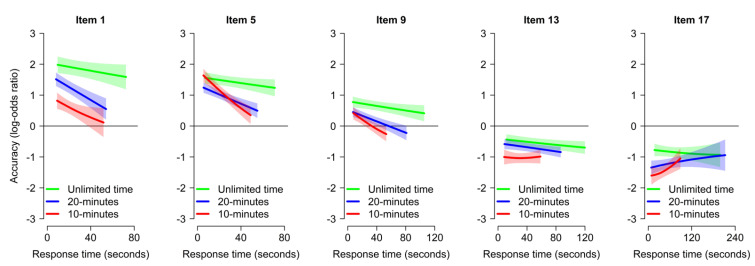
Accuracy (expressed as the log-odds ratio of a correct answer: 0 indicates a 50% chance of a correct answer) conditional on RT, for five items of the APM. For each condition, trajectories are plotted for the range of RTs comprising 95% of participants. Confidence bands represent +/−1 standard error.

To determine whether the relation between accuracy and RT was affected by time pressure, the effect of RT on accuracy was analyzed at the item level (with GAMMs including the main effect of RT, the main effect of item serial position, and the interaction between the two, as a function of time pressure). The results are represented in Figure 5.

The main effect of RT on accuracy was significant in the 20 min condition (χ^2^ = 5.66, *edf* = 1.00, *p* = 0.017) and marginally significant in the 10 min condition (χ^2^ = 6.15, *edf* = 1.75, *p* = 0.053), indicating that slower RTs were associated with a lower accuracy on average; there was no main effect for the Unlimited time condition (χ^2^ = 1.82, *edf* = 1.34, *p* = 0.318). The main effect of item position was significant in all conditions (all *p*s < 0.001), reflecting lower accuracy for later and more difficult items as expected. The two-way interaction between item position and RT was significant in the 20 min condition (χ^2^ = 6.34, *edf* = 1.10, *p* = 0.023) and in the 10 min condition (χ^2^ = 10.62, *edf* = 2.76, *p* = 0.028), reflecting the fact that the relation between RT and accuracy was less negative or even positive for the more difficult items, in line with the literature. There was no two-way interaction for the Unlimited time condition (χ^2^ = 0.38, *edf* = 1.00, *p* = 0.537).

Critically, the main effect of time pressure was significant (all *p*s < 0.001), indicating lower accuracy with increasing time pressure, regardless of participant RT; moreover, time pressure did not interact with the effect of RT (all *p*s > 0.363). As is visible in Figure 5, participants were overall less accurate under time pressure, and this was true for all items independently of their RT. In other words, the detrimental effect of time pressure on accuracy was not only attributable to speeding.

Of secondary interest, the two-way interaction between RT and item position differed between the 10 min condition and the Unlimited time condition (χ^2^ = 4.28, *p* = 0.039), indicating that the relation between RT and accuracy became more positive with increasing item difficulty in the 10 min condition than in the Unlimited time condition. This interaction may conceivably reflect a confound with ability (with only participants with high ability proceeding through the task quickly enough to have enough remaining time for slow RTs over the most difficult items in the 10 min condition). The same interaction did not differ between the 10 min condition and the 20 min condition (χ^2^ = 1.16, *p* = 0.282) or between the Unlimited time condition and the 20 min condition (χ^2^ = 1.41, *p* = 0.528).

### 3.5. Individual Differences and Time Pressure at the Task Level

The effect of time pressure on individual differences was first examined in terms of bivariate correlations between indices of performance in the APM and three measures of individual differences: ability (total performance in the APM, standardized separately within each condition), working memory (WMC), and motivation (NFC). Correlations, as summarized in Table 5, did not show a massive difference as a function of time pressure (Fisher’s *r* to *z* test only showed a significant decrease with time pressure for the relation between RT and individual differences and a somewhat higher correlation with confidence in the 20 min condition).

**Figure 6:**
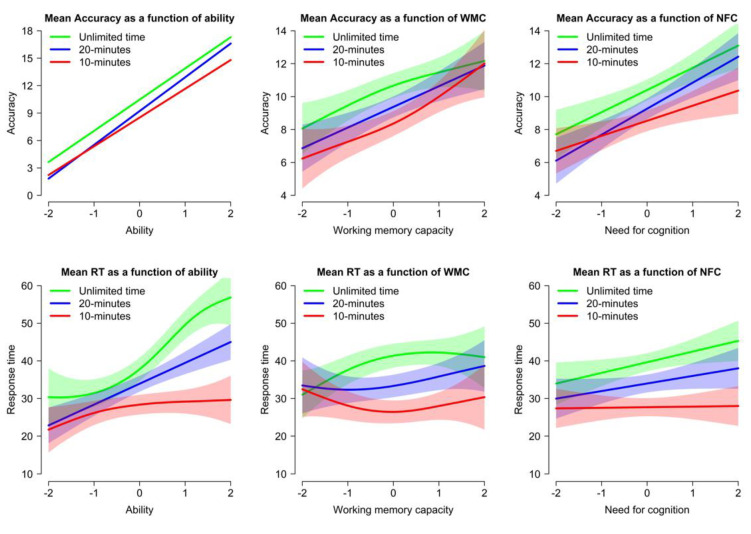
Effect of ability, WMC, and NFC on mean accuracy and RT. All predictors are standardized. Confidence bands represent +/−1 standard error.

Given the possibility that time pressure disproportionately affects high-performing participants, a better way to test this is to model the nonlinear relationships between performance and individual differences using GAMs (including the main effect of a given predictor, as a function of time pressure). This also offers a powerful way to determine whether time pressure could increase or decrease the distance between high-performing and low-performing participants, by examining predicted values. The results are represented in Figure 6, with the analyses detailed in Table 6 for accuracy and Table 7 for RTs.

For accuracy, the effect of individual differences on performance did not substantially change as a function of time pressure. There was a significant effect of ability, WMC, and NFC on accuracy in all conditions (with a beneficial effect on accuracy in all three cases), but there were no two-way interactions with condition. There were descriptively some differences (for example, the difference in predicted accuracy between a participant with NFC +2 SD vs. −2 SD from the mean was 5.39 points in the Unlimited time condition, but only 3.66 points in the 10 min condition), but these were not significant and displayed no consistent pattern. In sum, the results were not compatible with a differential effect of time pressure, contrary to part of the literature.

For RTs, the effect of individual differences on performance differed as a function of time pressure. Ability had a significant effect on RTs (with a higher ability being associated with slower RTs) in the Unlimited time and 20 min conditions, but not in the 10 min condition; WMC and NFC had a significant effect on RTs (with a higher WMC or NFC being associated with slower RTs) only in the Unlimited time condition. In other words, participants with higher ability, working memory, or motivation spent longer on APM problems, but this relation tended to decrease under time pressure. Contrasts between conditions were significant for ability (with significantly less effect of ability on RTs in the 10 min condition compared to the 20 min and Unlimited time conditions) and for WMC (with significantly less effect of WMC on RTs in the 10 min condition compared to the Unlimited time condition). The effect was in the same direction for NFC (e.g., participants with NFC +2 SD from the mean spent over eleven seconds more on APM problems than participants with NFC −2 SD from the mean in the Unlimited time condition, but there was less than one second of difference in the 10 min condition) but did not reach significance. In sum, there was a form of hard fall effect under time pressure with high-ability, high-WMC, and to an extent high-NFC participants being affected to a greater extent, but this was true only for RTs.

### 3.6. Individual Differences in RT modulation and Time Pressure at the Item Level

**Figure 7:**
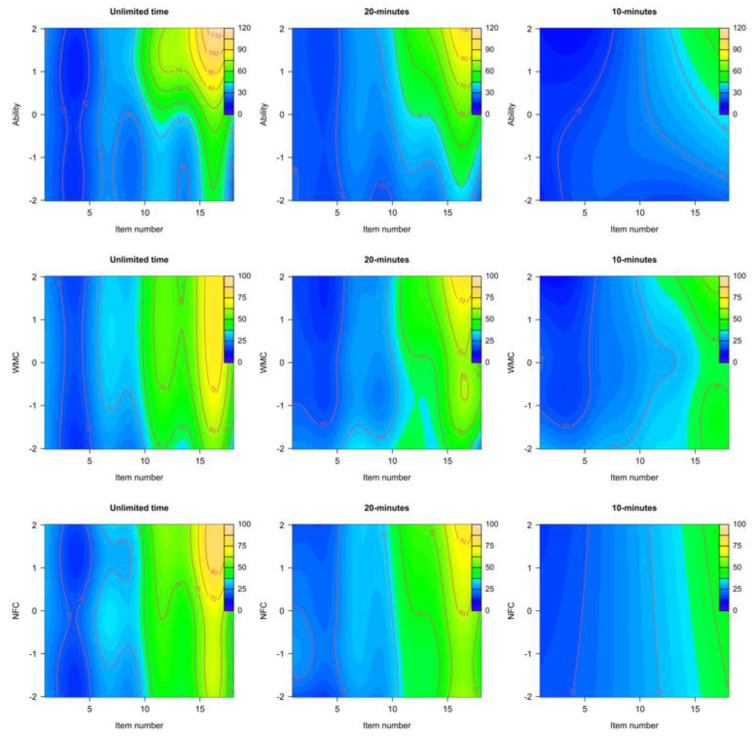
Modulation of RTs across item positions as a function of condition (columns) and individual differences in ability, WMC and NFC (rows).

Given the effect of time pressure on the relation between individual differences and RTs at the task level, an additional analysis was performed to test how the differences of modulation of RTs by individual differences as a function of time pressure unfolded at the item level. This was conducted using GAMMs (including the main effect of a given predictor, the main effect of serial position, and the interaction between the two, as a function of time pressure). Statistical tests are summarized in Table 8, with the results displayed in Figure 7. RTs in this figure are represented with colors ranging from blue (fast RT) to yellow (slow RT), with item position on the x-axis and individual differences on the y-axis. Overall, the results showed that the effect of time pressure on the modulation of RTs by individual differences differed as a function of item position.

The pattern was similar for individual differences in ability, WMC, and NFC. In all cases, time pressure made little difference for early items in the task, which had fast RTs in all conditions and regardless of individual differences. Instead, time pressure selectively affected RTs for individuals with a high ability, a high WMC, or a high NFC. In the Unlimited time condition, these individuals displayed significantly slower RTs for difficult items, reflecting modulation of effort in the face of difficulty (see Gonthier and Roulin 2020; Perret and Dauvier 2018). This modulation was slightly less pronounced in the 20 min condition and mostly disappeared in the 10 min condition (as reflected in both lower effect sizes and lower effective degrees of freedom indicating less non-linearity in the relation between individual differences and RT as a function of item position). In other words, time pressure selectively interfered with the modulation of RTs by high-ability, high-WMC, and high-NFC participants over difficult items.

## 4. Discussion

The major findings of this experiment with the effect of time pressure on response processes in Raven’s matrices can be summarized as follows:

1. Participants solved between 1 and 1.5 items per minute without time pressure. Mild and high time pressure induced speeding throughout the task, without a catch-up on later item positions, despite the fact that the moderate time pressure condition allowed enough time for virtually all participants to complete all items even without speeding. Participants did not use all the available time under time pressure: the average participant finished with 50% of time left under a mild pressure and 20% of time left under a high time pressure. Most participants attempted all items even under high time pressure, but a minority spent all their available time on early items.
2. Time pressure, even as mild as in the 20 min condition, significantly decreased accuracy, RTs, confidence in one’s answers, and the use of a constructive matching strategy. Time pressure did not significantly affect the use of a response elimination strategy or the metacognitive estimation of one’s accuracy.
3. Time pressure decreased accuracy, confidence in one’s answers, and the use of constructive matching relatively uniformly across all item positions. Time pressure decreased RTs significantly more for later items in the task, i.e., those items with higher difficulty and which usually require more time for correct completion.
4. Even mild time pressure induced significant or marginally significant speeding for all but two items in the task; in particular, there was significant speeding starting with the very first item. This speeding translated into a shift of the RT distribution for the whole sample towards faster RTs. Moderate time pressure had RTs closer to the high time pressure condition for the first half of the task and closer to the unlimited time condition for the second half.
5. There was an effect of both mild and high time pressure on accuracy conditional on RTs; in other words, time pressure decreased accuracy regardless of participant RT, which means lower accuracy under time pressure was not solely due to speeding. The relation between RT and accuracy was somewhat negative but tended towards positive for more difficult items, especially under time pressure.
6. The relationship between accuracy and individual differences in intellectual ability, working memory (WMC), or motivation (NFC) did not substantially vary as a function of time pressure. However, the relationship between RTs and individual differences was affected: individuals with higher ability, WMC, or NFC had slower RTs, but this difference tended to disappear under a time pressure for ability, WMC (significantly), and NFC (descriptively).
7. The effect of individual differences on RTs varied as a function of item position. Participants with a high ability, WMC, or NFC had slower RTs specifically for more difficult items, but for all three predictors, this RT modulation tended to disappear under a high time pressure.

These results were associated with large effect sizes for accuracy, RTs, and confidence ratings. Post hoc power analyses (Faul et al. 2007) show that the study was adequately powered for all effects at the task level except the small effects regarding strategy use (achieved power 0.95 for accuracy, 0.99 for RTs, 0.99 for confidence, and 0.58 for constructive matching). The findings are discussed in the next sections in the context of the three major questions: the effect of time pressure on speededness, on performance, and on the effect of individual differences in Raven’s matrices.

### 4.1. Question 1: Time Pressure and Speeding in Raven’s Matrices

The first aspect of the results regarding RTs is that time pressure induced speeding, as expected. Unexpectedly, however, time pressure induced speeding for all participants (or more precisely, it shifted the whole distribution of RTs), throughout the whole task, for most items including the first. This was the case even with the very forgiving time limit of the 20 min condition. Participants did not substantially slow down even when they had available time left and did not use all of the allowed time. Again, these points all suggest that the effect of time pressure goes well beyond forcing participants to skip some items due to insufficient time. Instead, time pressure yields a global speeding for all participants on average, throughout the whole task. On a secondary note, there was a small amount of variability in terms of test-taking strategies (Goldhammer 2015; Semmes et al. 2011), with most participants completing the whole test and just a couple of participants running out of time long before the end.

The very broad effect of time pressure on participant speeding is not attributable to participants carefully fine-tuning their time spent on an item as a function of available time left, as could be expected based on models proposing that the effect of time pressure mostly occurs for later items in the task (see also Bolsinova and Tijmstra 2015). This is all the more surprising that the early items actually do not require much time to be solved correctly. Two points seem worth mentioning here. The first is the role of test anxiety. The presence of a counter displaying remaining time in the two conditions with time pressure may have led to more stress regarding response times, encouraging participants to speed up more than required. This effect is likely, but difficult to test empirically: adding a counter of elapsed time to a condition with unlimited time would not induce the same pressure, and removing the counter would confound performance with the participants’ ability to estimate time and keep track of time.

The other important point is that participants taking the test do not have prior information regarding the amount of time that will be required for all items. This is obvious in the finding that participants in the 20 min condition finished on average with 50% of time left and sped up significantly compared to the Unlimited time condition, despite virtually all participants with unlimited time actually finishing under 20 min. The expectation that time pressure should only affect later items in the test implicitly assumes that participants have perfect information regarding the difficulty curve of the test and the typical RTs for an item. However, participants taking the test for the first time have no way to know how difficult later items will be and how much time they will require; therefore, it makes sense to speed up starting with the very first item, as a way to save time for later. Speeding up early may not be a good strategic decision given the relative difficulty of items presented at later serial positions, but participants also have no way to anticipate that the last items are so difficult that they are rarely solved correctly and no way to determine whether they have spent the optimal amount of time on the early items.

Based on these results, investigators interested in using a speeded version of the test for practical reasons may want to consider providing participants with information regarding the typical duration of the task. For example, instructions could inform participants (see Table 2) that "although they have 20 min to complete 18 problems, this is in fact sufficient for 95% of participants to complete the task comfortably at their own pace". This could help participants manage their time better and avoid the speeding behavior observed here, potentially limiting the detrimental effect of time pressure.

As expected, but contrary to the results for accuracy, time pressure disproportionately affected RTs for items presented at later serial positions: there was speeding for all items, but there was more speeding for items towards the end of the test. This would be expected based both on the fact that these more difficult items are more time-intensive and based on the fact that participants have less time remaining towards the end of the test. The surprising point, however, is that accuracy did not drop more for these items: participants speeded proportionally more on harder items, but their accuracy suffered to the same extent as for easier items, despite the relation between RT and accuracy tending towards neutral or positive for these items (in line with Becker et al. 2016; Dodonova and Dodonov 2013; Goldhammer et al. 2015). Performance in the later items of the APM was not too close to a floor effect (average performance for items in the last third of the task was still about 33% correct answers in the Unlimited time condition), so this was not due to a restriction of range preventing accuracy from going down further. Instead, this pattern may be due to the exponential increase in RTs observed for very difficult items without time pressure (Figure 3; see also Gonthier and Roulin 2020): it would seem that this spontaneous increase in RTs yields diminishing returns and that preventing participants from spending such a long time on difficult problems still allows them to provide a partial solution that keeps performance above guessing levels.

### 4.2. Question 2: Time Pressure and Performance in Raven’s Matrices

Time pressure naturally led to lower accuracy in Raven’s matrices, as could be expected. A more unexpected finding is that time pressure significantly decreased accuracy even in the 20 min condition (see Figure 2), which matched the usual time limit for Set II of Raven’s APM (20 min for 18 items or 40 min for the full 36 items) and which was sufficient for virtually all participants to complete the task without time pressure. Moreover, time pressure substantially decreased accuracy conditional on RTs: in other words, even participants with the same RT had lower accuracy on average under time pressure (see Figure 5). Another critical finding is that time pressure decreased accuracy uniformly throughout the task (including the very first item; see Figure 3), rather than specifically for the final items, despite time pressure having more effect on RTs for the final items. These three points together suggest that the detrimental effect of time pressure on accuracy was not, in fact, due specifically to speededness for a given item or to skipping the last items due to insufficient time. Instead, the detrimental effect of time pressure appears to be due to a broader impact on cognitive processing.

There are a number of possible mechanisms that could explain the detrimental effect of time pressure regardless of the amount of time available, RT, or item serial position. Given the current results, it seems likely that participants lowered their decision threshold for responding to an item, leading to the joint finding of lower accuracy and lower confidence. This might conceivably have translated into a decrease in the process of verifying one’s answer before responding (Goldhammer and Klein Entink 2011; Klein Entink et al. 2009a; Kyllonen and Zu 2016).

It also seems likely that participants engaged in qualitatively different processing of item information, given that simple acceleration of processing should not have impacted accuracy conditional on RTs. This qualitative difference may have come in the form of filtration of information (selectively considering less information before making a decision: Ben Zur and Breznitz 1981; Johnson et al. 1993; Wright 1974) and/or in the form of changes of strategy, reflected in significantly lower constructive matching and potentially increased guessing. The effect of time pressure on constructive matching was limited in size, but a single-question measure of constructive matching is not necessarily very accurate (see Jastrzębski et al. 2018), and guessing was not directly assessed.

A decrease in rule learning (Chuderski 2016) is a less likely contributor here, as it should have led to disproportionately lower accuracy for items presented later in the test. An effect of time pressure on test anxiety or motivation is possible, as these constructs were not assessed here. Regardless, the fact that metacognitive calibration did not vary as a function of time pressure suggests that participants were aware of the detrimental effect of time pressure, which means these changes of processing may conceivably represent conscious adaptations in the face of time constraints.

These findings regarding accuracy have several practical implications. First, they confirm that results obtained under a time pressure do not reflect the maximal performance of a participant (Raven 2008) and tend to underestimate the number of problems they are capable of solving, even if the amount of time allowed is very lenient. Second, imposing a time limit should be strongly discouraged for studies interested in measuring spontaneous variability in strategy use (e.g., Jastrzębski et al. 2018), in line with prior literature regarding high-level cognition (e.g., Friedman and Miyake 2004; Lépine et al. 2005; St Clair-Thompson 2007). Third, modeling the effect of speed or test speededness based selectively on items presented in later serial positions (e.g., Borter et al. 2020; Estrada et al. 2017; Schweizer and Ren 2013) is not advisable, given that early items are also affected by time pressure to a similar extent. Similarly, indices of item speededness based on the number of participants not reaching a given item due to time pressure (e.g., Stafford 1971) provide fundamentally flawed estimates of the “effect of speededness”, at least in the context of an intelligence test such as Raven’s APM.

### 4.3. Question 3: Time Pressure and Individual Differences

Time pressure had a limited detrimental effect on the ability of the APM to measure individual differences. For accuracy, the task had reliability below the conventional 0.70 threshold when performed under severe time pressure, which is in line with prior literature (Hong and Cheng 2019; Poulton et al. 2022) despite the difference in internal consistency not reaching significance. Importantly, reliability was significantly affected for RTs and also fell below 0.70 under severe time pressure. This suggests that imposing a time pressure makes the task counterintuitively less suitable for the assessment of response speed, possibly because of the additional variance in test-taking strategies under time pressure (Goldhammer 2015; Semmes et al. 2011; van der Linden 2009) or simply because time pressure interferes with the participants’ self-regulation of response speed, yielding more unstable RTs across items.

Besides measurement precision, time pressure did not substantially affect the relation between accuracy and cognitive ability (WMC) or motivation (NFC), either in terms of bivariate correlations or modeled as a nonlinear relationship. This indicates that time pressure did not have a major impact on the rank-ordering of participants (in line with Hamel and Schmittmann 2006; Preckel et al. 2011; Vernon et al. 1985; Wilhelm and Schulze 2002). Critically, the results also showed that time pressure had little impact on the distance between low- and high-performing participants (Figure 6). In other words, time pressure did not have a critical impact on the ability of the APM to measure individual differences: there was neither a major benefit (contrary to the prediction that the relation between performance and WMC should increase: Chuderski 2013, 2015; Tatel et al. 2020; but in line with the null results of Colom et al. 2015) nor a major drawback (contrary to the predictions of the choking under pressure account: Colom et al. 2015; Gimmig et al. 2006). It is possible that such effects do occur, but heavily depend on the precise mix of sample ability, task difficulty, and degree of speededness.

On the other hand, time pressure disproportionately affected RTs on difficult problems for participants with a high ability, a high WMC, and, to an extent, a high NFC. Although this did not directly translate into an effect on accuracy in the current study, this suggests that in some cases, time pressure could selectively interfere with the high performance of these participants, in line with predictions related to the phenomenon of choking under pressure. This point, along with the overall detrimental effect of time pressure on accuracy, suggests that time pressure should be avoided when assessing participants expected to demonstrate a high level of performance. For example, time pressure seems to be a risky choice in the context of giftedness assessment or highly selected samples with high ability overall.

Based on these results, it seems that the APM can be used in general to measure individual differences with a time constraint. While an unspeeded version will be a generally better choice, for investigators working with severe practical constraints related to the length of the testing session, imposing a time pressure may still be a better option than using highly shortened versions of the task (e.g., Hamel and Schmittmann 2006): very short versions can cause other issues such as low reliability due to less items and a different learning curve due to having less items to understand the rules before proceeding to more difficult items (for an example, see Ibrahim and Kazem 2013). However, this conclusion requires three caveats: increasing time pressure too much will also yield low reliability, for accuracy and especially for RTs; time pressure will lead to faster RTs, lower accuracy, and poorer strategy use than would have been observed on a shortened task, and time pressure will disproportionately affect the behavior of high-performing participants, especially on difficult items.

In sum, time pressure is not a universally better solution for the assessment of individual differences: it will work well when testing a sample with moderate ability, when the study is exclusively interested in rank-ordering rather than absolute levels of performance, and when the study is exclusively interested in performance rather than in the response processes leading to an answer (including response speed, test-taking strategies, etc). In other words, the APM can be safely speeded in the case where reasoning ability is to be used as a covariate in a broader study, rather than as the main focus of analysis.

### 4.4. Limitations and Future Directions

Three major questions were not explored in the present study. First, it would be interesting for future research to provide a more detailed look into the qualitative changes of processing that can occur under time pressure. Drift diffusion modeling is rarely used for this type of task and could be an interesting option (Frischkorn and Schubert 2018; Kang et al. 2022; Lerche et al. 2020; van der Maas et al. 2011), although this would not be straightforward and a larger dataset than was collected here would presumably be needed. Alternatively, verbal reports may be a good option for that purpose and could yield more insight into variability in time management strategies. Second, it would be worth examining the effects of time pressure on test anxiety and test motivation, as potential mediators of the detrimental effect of time pressure on performance. This is one of the major possible effects of time pressure as discussed here, and the one that has been least studied in the context of intelligence tests. Third, the experiment only examined the effects of time pressure at the task level, not at the item level (e.g., Kyllonen et al. 2018). Investigating item-level time pressure in the APM is less straightforward, because items of different difficulties have very different RTs (see Table 4, Figure 4). This calls for different time limits, given that applying the same moderate time limit to all items could result in easy items becoming practically unspeeded and difficult items becoming practically unsolvable. An experiment with variable time pressure at the item level could be interesting, although a preexisting dataset (such as the one provided here) would be necessary to calibrate appropriate time limits.

The results presented here heavily depend on the relations between ability, speed, and difficulty. For this reason, it is difficult to determine to what extent they are generalizable to other samples. University students in France are not an extremely biased sample (e.g., they do not undergo explicit selection based on their abilities), but they are still on average somewhat above the ability level of a community sample. It is possible that a sample with lower ability would be less affected by time pressure, due to time pressure having more effect on RTs for difficult items and high-performing participants. On the other hand, university students may be more used to working under time pressure, and there could conceivably be more strategic variability regarding time-on-task in a more diverse sample. Moreover, this study only considered individual differences in young adults: other populations may have different ways of coping with time pressure. Although some studies have found that the pattern of age differences does not substantially vary as a function of speededness in older adults (Babcock 1994), the effect of time pressure can interact with developmental differences, artificially inflating differences between younger and older children (Borter et al. 2020). A dedicated study of how response processes interact with characteristics of the sample would be enlightening.

Likewise, the results may differ with other task conditions. One example is compositional versions of the task (such as Duncan et al. 2017), where participants have to draw or construct their own answer, removing the possibility of proceeding by response elimination. Time pressure may be undersirable in this case due to the items requiring time to construct an answer, possibly with individual differences. Another example is that there are other versions of Raven’s matrices with different arrangements of item difficulties. In particular, Raven’s Standard Progressive Matrices (SPM) comprise five sets of twelve problems with difficulty arranged in a wave-like pattern (e.g., difficulty increases across the twelve items of set A; item B01 is less difficult than item A12 but more difficult than item A01). Contrary to the APM, where difficulty is confounded with serial position, this design means difficulty does not increase linearly throughout the task. There are indications that this may generate less participant disengagement over very difficult items (Gonthier and Roulin 2020; see also Perret and Dauvier 2018), and it could also affect the way participants dynamically manage their RTs under time pressure: contrary to the APM, the probability of a correct answer can increase from one item to the next, which means it may be profitable to selectively increase and decrease RT throughout the task.

Another interesting extension would be to test the effects of time pressure for other types of high-level cognitive tasks altogether, as the same questions broadly apply (for an example with creativity, see Preckel et al. 2011). As can be seen with the present work, time pressure has complex effects of response processes at the item level as a function of individual differences, some of which are difficult to predict. The extent to which this affects results is unknown for the vast majority of tasks and settings, which creates a potential source of inconsistency across studies for all types of high-level cognitive tasks and constructs.

## Figures and Tables

**Table 1:** Descriptive statistics for all measures at the task level, as a function of condition.

Condition	Measure	*M*	*SD*	Skew	Kurtosis	Range	α
Unlimited time (*n* = 97)	Accuracy	10.47	3.43	0.02	−0.47	2–18	0.74
	Response time	39.80	15.50	1.15	1.77	13.21–101.23	0.85
	Confidence	63.00	14.43	0.12	−0.82	32.72–93.44	0.85
	Constructive matching	6.75	1.69	−0.98	0.51	1.67–9	0.94
	Response elimination	5.78	1.74	−0.53	−0.47	1.44–8.50	0.92
	Strategy use	0.96	2.58	−0.06	0.91	−6.16–7.11	0.92
20 min time pressure (*n* = 99)	Accuracy	9.22	3.71	0.15	−0.49	1–18	0.77
	Response time	33.94	12.32	0.69	0.36	10.58–69.43	0.81
	Confidence	56.37	19.25	−0.05	−0.63	12.78–94.56	0.92
	Constructive matching	6.34	1.67	−0.40	−0.41	2.06–9	0.93
	Response elimination	5.51	1.63	−0.37	−0.49	1.11–8.78	0.92
	Strategy use	0.83	2.45	0.41	0.07	−4.94–7.66	0.92
10 min time pressure (*n* = 99)	Accuracy	8.52	3.16	−0.13	−0.36	1–15	0.68
	Response time	27.69	7.54	1.84	10.55	10.63–72.05	0.65
	Confidence	52.05	17.03	−0.20	−0.16	8.78–96.17	0.89
	Constructive matching	6.19	1.61	−0.67	0.16	1–8.88	0.92
	Response elimination	5.53	1.57	−0.12	−0.71	2.17–8.78	0.90
	Strategy use	0.66	2.30	−0.31	0.64	−7.11–6.33	0.89

Note. Possible values range from 0 to 18 for accuracy, from 0 to 100 for confidence, and from 1 to 9 for strategy use; response times are expressed in seconds. The columns represent mean, standard deviation, skewness, kurtosis, range, and Cronbach’s alpha.

**Table 2:** Percentile ranks for time-on-task in the Unlimited time condition.

Percentile	Time-on-Task (Minutes)	Item Completion Rate (Items/Minute)
02.5%	06.23	2.89
05%	06.60	2.73
10%	06.97	2.58
25%	08.77	2.05
50%	11.29	1.59
75%	14.51	1.24
90%	18.45	0.98
95%	20.61	0.87
97.5%	21.43	0.84

Note. These values are for the 18 items of the APM used here: times would be different for the full 36-items version of the APM.

**Table 3:** ANOVAs for the effect of time pressure at the task level.

Measure	*F*(2, 292)	*p*	η^2^_p_	*HSD*
Accuracy	8.13	<0.001	0.05	(10 = 20) < UN
Response time	24.14	<0.001	0.14	10 < 20 < UN
Confidence	10.25	<0.001	0.07	(10 = 20) < UN
Constructive matching	2.99	0.050	0.02	10 < UN
Response elimination	0.85	0.428	0.01	ns
Strategy score	0.38	0.681	0.00	ns

Note. (10 = 20) < UN indicates that there was no significant difference between the 10 min condition and the 20 min condition, but both conditions were significantly lower than the Unlimited time condition.

**Table 4:** Comparison of mean RTs across conditions, at the item level.

Item	Mean RT in Seconds			ANOVA Results			
	Unlimited	20-min	10-min	*F*(2, 292)	*p*	η^2^	*HSD*
Item 01	29.80	20.70	21.37	12.99	<0.001	0.08	(10 = 20) < UN
Item 02	19.34	17.31	15.93	3.11	0.046	0.02	10 < UN
Item 03	17.53	15.46	14.82	2.61	0.075	0.02	ns
Item 04	21.57	20.46	19.74	0.52	0.592	0.00	ns
Item 05	24.61	20.34	18.80	3.90	0.021	0.03	10 < UN
Item 06	24.19	21.10	20.19	2.71	0.068	0.02	ns
Item 07	34.61	28.15	26.13	6.36	0.002	0.04	(10 = 20) < UN
Item 08	33.53	28.42	25.47	8.77	<0.001	0.06	(10 = 20) < UN
Item 09	29.22	26.17	23.44	2.09	0.125	0.01	ns
Item 10	39.43	27.28	26.51	6.18	0.002	0.04	(10 = 20) < UN
Item 11	48.27	36.24	30.59	17.32	<0.001	0.11	(10 = 20) < UN
Item 12	53.74	45.97	34.47	12.80	<0.001	0.08	10 < (20 = UN)
Item 13	41.61	35.88	28.49	9.79	<0.001	0.06	10 < (20 = UN)
Item 14	57.62	45.81	32.93	12.45	<0.001	0.08	10 < 20 < UN
Item 15	50.70	48.80	32.86	16.16	<0.001	0.10	10 < (20 = UN)
Item 16	72.70	62.93	44.53	11.74	<0.001	0.08	10 < (20 = UN)
Item 17	72.85	61.63	41.99	12.82	<0.001	0.08	10 < (20 = UN)
Item 18	45.12	47.68	36.40	3.42	0.034	0.02	10 < (20 = UN)

Note. (10 = 20) < UN indicates that there was no significant difference between the 10 min condition and the 20 min condition, but both conditions had significantly lower RTs than the unlimited time condition.

**Table 5:** Bivariate correlations between individual differences and APM performance as a function of time pressure.

Measure	Correlation with Ability			Correlation with WMC			Correlation with NFC		
	Free	20-min	10-min	Free	20-min	10-min	Free	20-min	10-min
Accuracy	1.00	1.00	1.00	0.32	0.34	0.42	0.39	0.43	0.30
Response time	0.51	0.45	0.24	0.19	0.14	-0.07	0.18	0.16	0.02
Confidence	0.53	0.65	0.29	0.25	0.54	0.30	0.36	0.47	0.28
Constructive matching	0.30	0.31	0.28	0.19	0.30	0.23	0.34	0.30	0.36
Response elimination	−0.36	−0.16	−0.36	−0.24	−0.19	−0.26	−0.29	−0.17	−0.28

Note. Pearson’s *r* correlation coefficients.

**Table 6:** Effect of individual differences on accuracy as a function of time pressure.

Test	Condition	Predictor		
		Ability	WMC	NFC
Main effect of predictor on accuracy	Unlimited	-	*F* = 7.29, *edf* = 1.52, *p* = 0.003	*F* = 16.42, *edf* = 1.00, *p* < 0.001
	20 min	-	*F* = 14.83, *edf* = 1.00, *p* < 0.001	*F* = 23.81, *edf* = 1.00, *p* < 0.001
	10 min	-	*F* = 8.47, *edf* = 1.64, *p* < 0.001	*F* = 8.43, *edf* = 1.00, *p* = 0.004
Difference between −2/+2 SD	Unlimited	13.65	4.11	5.39
	20 min	14.78	5.03	6.32
	10 min	12.58	5.77	3.66
Difference between conditions	Unlimited vs. 20 min	-	*F* = 0.20, *p* = 0.658	*F* = 0.25, *p* = 0.618
	Unlimited vs. 10 min	-	*F* = 0.73, *p* = 0.517	*F* = 0.89, *p* = 0.347
	20 min vs. 10 min	-	*F* = 0.54, *p* = 0.628	*F* = 2.16, *p* = 0.143

Note. “Difference between −2/+2 SD” refers to the difference in predicted values of accuracy for a participant with a predictor value −2 SD or +2 SD away from the mean; for example, in the Unlimited time condition a participant with ability +2 SD from the mean would be predicted to perform 13.65 points higher than a participant −2 SD from the mean.

**Table 7:** Effect of individual differences on RTs as a function of time pressure.

Test	Condition	Predictor		
		Ability	WMC	NFC
Main effect of predictor on RTs	Unlimited	*F* = 15.48, *edf* = 2.94, *p* < 0.001	*F* = 3.42, *edf* = 2.03, *p* = 0.024	*F* = 5.08, *edf* = 1.00, *p* = 0.025
	20 min	*F* = 26.42, *edf* = 1.00, *p* < 0.001	*F* = 1.31, *edf* = 1.69, *p* = 0.295	*F* = 2.71, *edf* = 1.00, *p* = 0.101
	10 min	*F* = 1.73, *edf* = 1.82, *p* = 0.148	*F* = 0.92, *edf* = 1.96, *p* = 0.364	*F* = 0.02, *edf* = 1.00, *p* = 0.896
Difference between −2/+2 SD	Unlimited	26.47	9.98	11.34
	20 min	22.17	5.20	8.06
	10-min	7.92	−2.10	0.63
Difference between conditions	Unlimited vs. 20 min	*F* = 2.00, *p* = 0.089	*F* = 0.68, *p* = 0.559	*F* = 0.22, *p* = 0.640
	Unlimited vs. 10 min	*F* = 6.30, *p* < 0.001	*F* = 3.21, *p* = 0.022	*F* = 2.38, *p* = 0.123
	20 min vs. 10 min	*F* = 3.08, *p* = 0.035	*F* = 1.46, *p* = 0.200	*F* = 1.18, *p* = 0.278

Note. “Difference between −2/+2 SD” refers to the difference in predicted values of RT for a participant with a predictor value −2 SD or +2 SD away from the mean; for example, in the Unlimited time condition a participant with ability +2 SD from the mean would be predicted to respond 26.47 seconds slower than a participant −2 SD from the mean.

**Table 8:** Interaction between individual differences and item position for RTs as a function of time pressure.

Test	Condition	Predictor		
		Ability	WMC	NFC
Interaction between predictor and item position	Unlimited	*F* = 21.61, *edf* = 10.88, *p* < 0.001	*F* = 6.84, *edf* = 2.15, *p* < 0.001	*F* = 6.54, *edf* = 7.71, *p* < 0.001
	20 min	*F* = 20.28, *edf* = 5.08, *p* < 0.001	*F* = 4.58, *edf* = 8.96, *p* < 0.001	*F* = 7.83, *edf* = 3.26, *p* < 0.001
	10 min	*F* = 17.23, *edf* = 3.06, *p* < 0.001	*F* = 4.35, *edf* = 3.12, *p* = 0.007	*F* = 1.63, *edf* = 1.00, *p* = 0.202
Difference between conditions	Unlimited vs. 20 min	*F* = 3.41, *p* = 0.025	*F* = 1.91, *p* = 0.037	*F* = 2.19, *p* = 0.049
	Unlimited vs. 10 min	*F* = 12.80, *p* < 0.001	*F* = 1.75, *p* = 0.218	*F* = 5.71, *p* = 0.002
	20 min vs. 10 min	*F* = 5.09, *p* = 0.006	*F* = 3.85, *p* = 0.050	*F* = 4.35, *p* = 0.007

## Data Availability

The data reported in this study, as well as sample R code, are available on the Open Science Framework (OSF) at https://osf.io/9rtxf/ (uploaded 12 June 2023).

## Note

1 Response times on the concurrent processing tasks of each complex span can also be used as an estimate of mental speed: for an example, see Unsworth et al. (2009). However, given the complex nature of the processing tasks, this speed measure is also confounded with intellectual ability. Using this measure for the analyses of individual differences gave results closer to what could be expected with a measure of intellectual ability: participants with lower speed on the concurrent processing tasks were less accurate overall, but the effect of speed decreased under time pressure. These results are not detailed here.
